# Exogenous bone marrow derived-putative endothelial progenitor cells attenuate ischemia reperfusion-induced vascular injury and renal fibrosis in mice dependent on pericytes

**DOI:** 10.7150/thno.48562

**Published:** 2020-10-25

**Authors:** Meng Wang, Huzi Xu, Yinzheng Li, Chujin Cao, Han Zhu, Yuxi Wang, Zhi Zhao, Guangchang Pei, Fan Zhu, Qian Yang, Xuan Deng, Cheng Zhou, Yi Guo, Jianliang Wu, Wenhui Liao, Juan Yang, Ying Yao, Rui Zeng

**Affiliations:** 1 Nephrology, Tongji Hospital, Tongji Medical College, Huazhong University of Science and Technology, 1095 Jiefang Ave, Wuhan, Hubei, 430030, China.; 2 Geriatrics, Tongji Hospital, Tongji Medical College, Huazhong University of Science and Technology, 1095 Jiefang Ave, Wuhan, Hubei, 430030, China.; 3 Nutrition, Tongji Hospital, Tongji Medical College, Huazhong University of Science and Technology, 1095 Jiefang Ave, Wuhan, Hubei, 430030, China.

**Keywords:** PDGF-BB/PDGFR-β, pericyte, pEPCs, vascular injury, renal fibrosis.

## Abstract

**Rationale:** Capillaries are composed of endothelial cells and the surrounding mural cells, pericytes. Microvascular repair after injury involves not only the proliferation of endothelial cells but also pericyte-based vessel stabilization. Exogenous bone marrow derived-putative endothelial progenitor cells (b-pEPCs) have the potential for vascular repair; however, their effect on vascular structure stabilization and pericyte-related pathobiological outcomes in the injured kidney has not been fully examined.

**Methods:** We applied ischemia-reperfusion (IR) to induce renal vascular injury and renal fibrosis in mice. Platelet-derived growth factor receptor β (PDGFR-β)-DTR-positive mice were generated to deplete pericytes, and exogenous b-pEPCs and the PDGFR-β ligand, PDGF chain B (PDGF-BB), were employed to explore the relationship among b-pEPCs, pericytes, vascular repair, and early renal fibrosis.

**Results:** Administration of b-pEPCs reduced IR-induced pericyte-endothelial detachment, pericyte proliferation, and myofibroblast transition via a paracrine mode, which preserved not only vascular stabilization but also ameliorated IR-initiated renal fibrosis. PDGF-BB upregulated the expression of PDGFR-β, exacerbated vascular abnormality, and pericyte-myofibroblast transition, which were ameliorated by b-pEPCs administration. The exogenous b-pEPCs and their culture medium (CM) induced vascular injury protection, and renal fibrosis was blocked by selective deletion of pericytes.

**Conclusion:** Exogenous b-pEPCs directly protect against IR-induced vascular injury and prevent renal fibrosis by inhibiting the activation of PDGFR-β-positive pericytes.

## Introduction

Ischemia-reperfusion (IR) is one of the common forms of kidney injury 1, which creates a microenvironment with oxygen and nutrient deficiency, increased oxidative stress, and reactive oxygen species accumulation, leading to endothelial cell apoptosis, necrosis, and injury 2, 3. Inflammatory and chemokine factors are generated and released into the renal interstitium, stimulating mesangial cell proliferation and production of extracellular matrix (ECM), leading to chronic renal injury and fibrosis. The increased number of mesangial cells and ECM occupy large interstitial space, shrink the capillaries, inhibit endothelial cell renewal, and increase the oxygen transfer distance from capillaries to renal epithelial cells, which in turn leads to more aggressive anoxia, a sustained inflammatory or growth factor release and exacerbates vascular injury and fibrosis 4-6. Angiogenesis is a process in which new endothelial cells and vessels are formed for vascular renewal or in response to injury. Early vascular regeneration provides enough oxygen and nutrients to reduce oxidative stress and reactive oxygen species accumulation, promote self-repair, and preserve renal function 7, 8. Thus, previous research mostly focused on early and effective angiogenesis by promoting endothelial cell proliferation, such as, by stem cell transplantation 9, 10.

Besides endothelial cell proliferation, vascular structure stabilization also plays an important role in vascular preservation and repair. Capillaries consist of endothelial cells that connect arterioles with venules and form networks covered by pericytes, providing structural support for endothelial cells to sustain vessel integrity and permeability by crosstalk or paracrine pathways 11-14. The association between pericytes and endothelial cells provides new insights into the microvasculature architecture 15, suggesting potential therapeutic strategies for vascular preservation. Pericyte proliferation restores damaged endothelial cells, and the maturation of mural cell pericytes has been reported to enhance their attachment to endothelial cells for stabilization and formation of functional capillaries 16, 17.

Putative endothelial progenitor cells (pEPCs) are hematopoietic stem cells that can differentiate into endothelial cells and have been shown to participate in angiogenesis in different organs 18, 19. In our previous study, we observed a protective effect of exogenous bone marrow derived-pEPCs (b-pEPCs) on unilateral ureteral obstruction (UUO)-induced renal fibrosis, independent of their capacity to differentiate into endothelial cells, but by inhibiting pericyte-myofibroblast transition 20. However, definitive evidence was needed to confirm the ability of exogenous b-pEPCs to access and act on renal pericytes in injured kidneys. In this study, we used pericyte genetic depletion mice to investigate whether the angiogenic capacity of exogenous b-pEPCs has intrinsic links with pericytes. Our findings conclusively established that exogenous b-pEPCs prevented pericyte-endothelial cell detachment, pericyte proliferation, and transition to myofibroblast via a paracrine pathway, thus attenuating vascular structure instability and subsequent renal fibrosis.

## Methods

### Mice and cells

Eight-week-old male wild-type (WT) mice were purchased from Beijing Huafukang Laboratory Animal Technology Co., Ltd., Beijing, China. IR surgery and b-pEPCs treatment was performed following a previously described procedure 20. In our previous study, we referred to some paper and tried different ischemia durations include 25min, 30min and 45min. Mice with a 25min duration of unilateral ischemia reperfusion showed mild tubular injury at day 1 and 2, some reversed to normal at day 5. Mice with 45min showed rather serious tubular injury and fibrosis than 30min, but the mortality in 24h after surgery increased too. As a compromise, we adopted a 30min duration of ischemia reperfusion injury (Data not shown). The left kidney was chosen for IR surgery and the left renal artery was clamped for 30 min 21. When the clamp was removed to start reperfusion, the left kidney reverted from black to red color within approximately 10 s. Throughout the surgery, animal body temperature was maintained at 36.6-37.2 °C with a temperature control machine (FHC, USA) 20, 22. GFP-b-pEPCs were obtained from CAG-enhanced green fluorescent protein (EGFP) mice (JAX #006567, Jackson Laboratory, USA). All experiments were approved by the Animal Care and Use Committee of Tongji Hospital. b-pEPCs derived from WT murine bone marrow were cultured in FBS-free culture medium for 24 h. The b-pEPCs culture medium (b-pEPCs-CM) was collected after filtration through a 0.22 μm filter. In the first part of experiment, mice were randomly divided into various groups with 3-5 mice in each group: sham-operated mice (sham), sham-operated mice treated with b-pEPCs (b-pEPCs), mice subjected to IR surgery treated with (IR+b-pEPCs) or without b-pEPCs (IR). In the second part, sham-operated mice were treated with b-pEPCs-CM (b-pEPCs-CM), IR mice were treated with b-pEPCs-CM (IR+b-pEPCs-CM), or control vehicle culture medium EBM-2 (IR+EBM-2).

DTR-PD mice were generated by crossing iDTR mice (Jackson Laboratory, USA) and PDGFR-β*^tm1(iCre)^*(B-CM-004, BioCytogen, China) mice. After induction with diphtheria toxin (DT, 50 ng/g, Sigma-Aldrich), PDGFR-β-DTR positive cells were depleted.

Human brain vascular pericytes (HBVP, Cat. #1200) were purchased from ScienCell Research Laboratories and cultured using the Pericyte Medium (PM, Cat. #1201) according to manufacturer's instructions.

### Bone marrow transplantation and conjoined parabiosis

Bone marrow transplantation (BMT): 8-week old male mice were anesthetized with 1% sodium pentobarbital (10 μL/g, Promoter, Wuhan, China) and irradiated with X-ray using RS2000 X-ray biological irradiator (300 rad/min with a total dose of 7.5 Gy). Bone marrow-derived cells (BMCs) were isolated from EGFP mice. Cells were collected and filtered. The red blood cells were lysed by incubating in a lytic solution for 5 min, and the remaining cells were collected as GFP-labeled BMCs (GFP-BMCs). 1*10^7^ GFP-BMCs were injected into mice via the tail vein.

Conjoined parabiosis: A WT mouse and an EGFP mouse were shaved following anesthesia, and a longitudinal back skin incision was made. Subsequently, the wounds were sutured together as previously described 23.

### PDGF-BB and TGF-β treatment

Mice were intraperitoneally injected with PDGF-BB (100 ng/mouse, Peprotech, USA) 1 and 24 h after the IR surgery. Pericytes were stimulated *in vitro* with PDGF-BB (10 ng/mL) or transformation growth factor-β (TGF-β) (10 ng/mL, R&D system, USA) for 24 h.

### Histologic and immunofluorescence staining

Renal samples were formalin-fixed, paraffin-embedded, sectioned at 3 μm, and processed for routine periodic acid Schiff (PAS) and Masson trichrome (Masson) staining. Tubular damage included tubular dilation, shape change of tubular cells from cuboid to flat, brush border injury, loss, cast formation, tubular cell death, and detachment. At least 10 high power fields of view in each kidney were examined. Immunofluorescence staining of α-smooth muscle actin (α-SMA, 1:100, Abcam), CD31 (1:100, BD), GFP (1:100, Abcam), proliferative cell nuclear antigen (PCNA, 1:200, CST), collagen 1α1 (1:100, Abcam), and PDGFR-β (1:50, Abcam) was performed at 4 ℃ overnight and labeled with secondary antigens. Nuclei were marked with 4′ 6-diamidino-2-pheny-lindole (DAPI). Staining was quantified in 6-10 randomly chosen fields on each slide and the data were analyzed using Image-Pro Plus software (Media Cybemetics, Rockville, MD, USA) in a blind manner.

### Western blotting

Renal and cellular protein samples were extracted by protein extraction kit (Promoter, Wuhan, China), and the protein concentration was quantified using the BCA Protein Assay (Promoter, Wuhan, China). Proteins were separated by SDS-PAGE and transferred to PVDF membranes (Millipore, Billerica, MA, USA). The membranes were blocked with 5% skimmed milk for 1 h at 37 °C and then probed with antibodies against α-SMA (1:5000, Abcam), PDGFR-β (1:3000, Abcam), Fibronectin (1:2000, Abcam), and GAPDH (1:4000, Abbkine) at 4 °C overnight. The blots were incubated with HRP-conjugated secondary antibodies. The signal intensities of target bands were quantified using Image J software (NIH, USA).

### Quantitative real-time PCR (qRT-PCR)

Total RNA was isolated from mouse renal tissues using Trizol reagent (Invitrogen, USA). cDNA reverse transcription was performed using primers and GoScript reverse transcription system (Promega, USA). qRT-PCR was performed with SYBR Green master mix (Qiagen, Germany) on Roche light 480II system. Results were normalized to GAPDH. The sequences of primers used are listed in Table 1.

### Flow cytometry

The kidney was diced and digested into single-cell suspension. Cells were first incubated with 5% bovine serum albumin (BSA, Sigma) for 15 min and then incubated with anti-mouse CD34-eFluor 660 (eBioScience) at room temperature for 1 h and analyzed using BD fluorescence-activated cell sorting (FACS) flow cytometry.

### Serum blood urea nitrogen (BUN) and creatinine measurement

BUN and creatinine were tested using test kits (Changchunhuili, China) according to the manufacturer's instructions.

### Tube-formation assay

Human umbilical vein endothelial cells (HUVECs) were seeded onto Matrigel-coated 96-well plates (10000 cells/well) according to manufacturer's instruction (BD Bioscience, USA). Plates were incubated in a humidified incubator (Thermo Fisher, USA) and images were taken at 0.5, 1, 2, and 8 h.

### Statistical analyses

GraphPad Prism 6.0 software was used for statistical analysis. Results were presented as mean ± SD, and all experiments were performed at least in triplicate. Statistical differences between two groups were analyzed with an unpaired *t* test or Mann-Whitney *U* test. Differences were considered statistically significant when *p*<0.05.

## Results

### Exogenous b-pEPCs protected mice from IR-induced renal injury and subsequent early fibrosis

**Figure 1:**
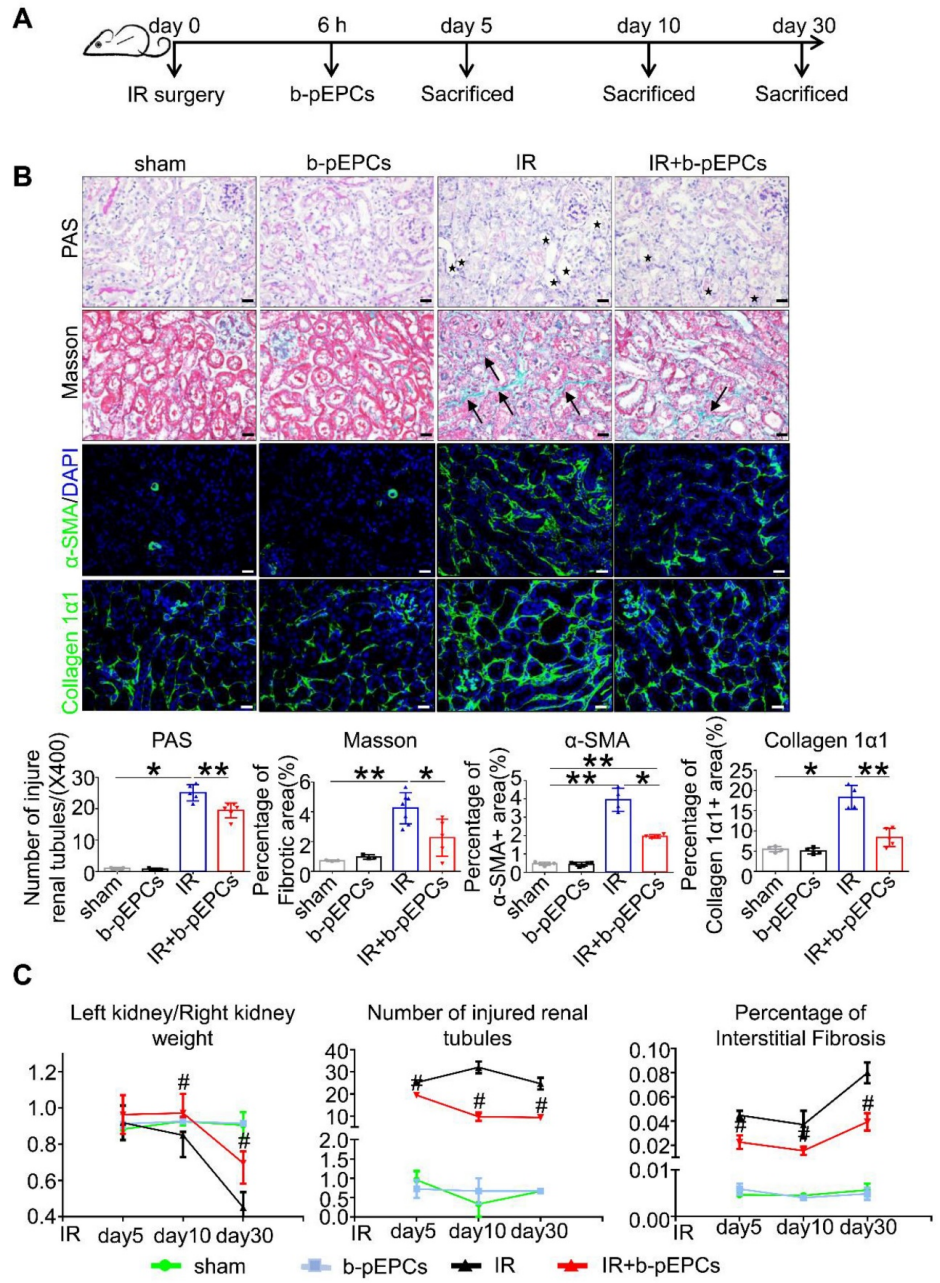
Exogenous b-pEPCs protected mice from IR-induced renal injury and early fibrosis.** A.** Experiment schematic.** B.** PAS, Masson, α-SMA and collagen 1α1 staining. PAS staining was performed to evaluate renal tubular injury by calculating tubules with dilation swelling or epithelial cell brush border loss (black stars). The fibroid green area in Masson staining (black arrows) indicates extracellular fibrotic collagen deposition in renal interstitium. α-SMA is a marker of myofibroblast, the α-SMA-labeled area was measured for evaluation of myofibroblast infiltration. Collagen 1α1 was used to evaluate collagen deposition in renal interstitium. **C.** Statistical histograms of Left kidney/Right kidney, tubular injury in PAS and fibrosis in Masson staining at different time-course. Data are presented as the mean ± SD, n=3-6/group, *p<0.05, **p<0.01; # represents a comparison between IR and IR+b-pEPCs group at same time course, *p*<0.05. Scale bar: 20 μm.

It has previously been reported that stromal cell-derived factor-1 (SDF-1), which recruits stem cells to injured tissues, increased and peaked at 6 h after injury 24. Therefore, we injected b-pEPCs into sham-operated mice (IR group) and in mice 6 h after IR surgery (b-pEPCs group) (Figure 1A). PAS staining showed no difference in injured tubule numbers between sham and b-pEPCs groups, indicating no additional damage to the normal kidney by b-pEPCs injection. In the IR group, massive renal tubules were dilated with epithelial cell brush border loss while in the IR + b-pEPCs group, the number of injured tubules decreased (*p*<0.01). Masson staining showed an enlarged fibrotic area in renal interstitium in the IR group (4.24%) compared with 2.26% in the IR + b-pEPCs group (Figure 1B). Immunofluorescence staining with α-SMA, a marker of myofibroblasts, showed that after IR surgery, a large number of α-SMA-positive cells appeared in renal interstitium, while b-pEPCs injection reduced the number of α-SMA-positive cells. Measurement of another fibrotic marker, collagen 1α1, showed more collagen deposition in renal interstitium in the IR group than IR+b-pEPCs group (Figure 1B).

Next, we prolonged the experiment time and observed improved tubular injury and interstitial fibrosis in IR+b-pEPCs group on days 10 and 30 after IR by PAS and Masson staining. As the *in situ* loss of parenchymal cells (including tubular epithelial cells, podocytes, etc.) and fibrotic collagen deposition led to a weight loss of the injured kidney, the “left kidney/right kidney weight” was adopted to evaluate renal fibrosis. The results showed that b-pEPCs administration improved the “left kidney/right kidney weight” ratio after IR injury (Figure 1C), demonstrating that exogenous b-pEPCs protected mice from IR-induced renal injury and subsequent early fibrosis.

### Exogenous b-pEPCs protected mice from IR-induced vascular injury and renal fibrosis via paracrine pathways

**Figure 2:**
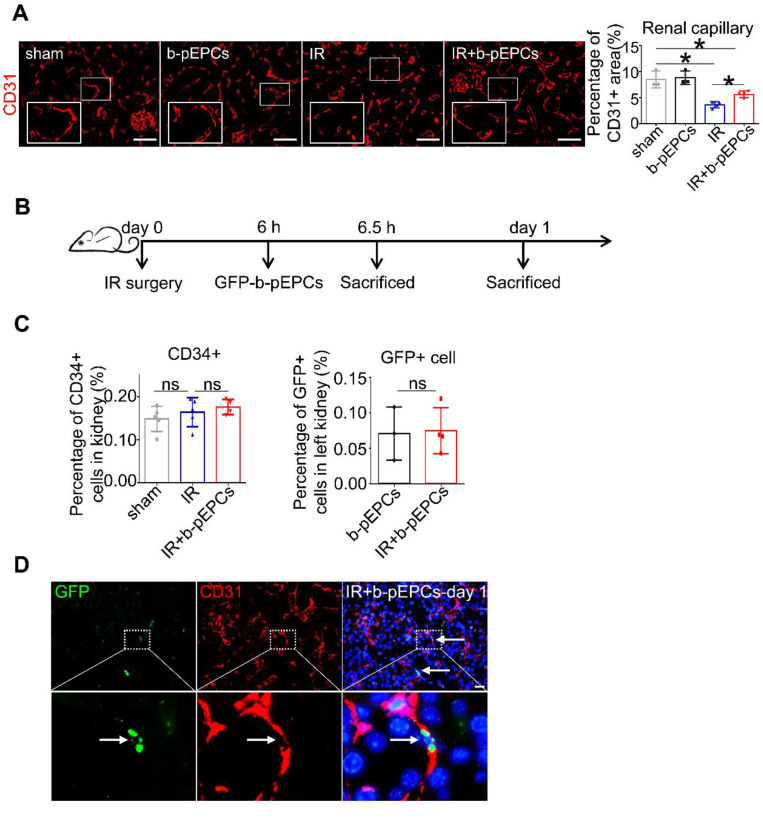
Differentiation pf exogenous GFP-positive b-pEPCs into endothelial cells was tracked. **A.** Immunofluorescence staining of CD31 to evaluate renal capillaries.** B.** Scheme of b-pEPCs tracking experiment.** C.** b-pEPCs isolated from EGFP mice were transplanted into mice after IR surgery. Both CD34-positive and GFP-positive cells were counted by flow cytometry to track exogenous b-pEPCs in the IR-operated kidney. **D.** Co-staining of GFP and CD31 were carried out to track GFP-b-pEPCs in the injured kidney and probable links with endothelial cells. Data are presented as the mean ± SD, n=3-5/group, *p<0.05, ns: no significant difference. Scale bar: 50 μm.

b-pEPCs may be considered hematopoietic stem cells due to their capacity to differentiate into endothelial cells. We detected renal interstitial capillaries with the endothelial cell marker CD31. Immunofluorescence staining showed that IR caused capillary rarefaction and structure breakage, while b-pEPCs injection attenuated the density loss of interstitial capillaries (Figure 2A). Co-staining of CD31 and PCNA indicated only a few endothelial cells in the healthy kidney were in proliferative status, whereas most of them were in the resting state (Figure S1). In the IR group, endothelial cells suffered damage and presented with poor proliferative capacity. By contrast, exogenous b-pEPCs administration increased the number of proliferative endothelial cells, angiogenesis, and vascular repair (Figure S1). We observed that peripheral blood cells directly differentiated into endothelial cells in bone marrow transplantation and conjoined parabiosis models (Figure S2) and hypothesized that b-pEPCs protected vascular injury by directly differentiating into endothelial cells and participating in angiogenesis. Thus, we tracked exogenous b-pEPCs using cells from EGFP-transgenic mice (Figure 2B). Flow cytometry of CD34, a special marker of b-pEPCs, showed no difference among sham, IR, and IR+b-pEPCs groups, indicating that the number of b-pEPCs recruited to the injured kidney did not increase after b-pEPCs injection. The number of GFP-positive cells in the IR+b-pEPCs and b-pEPCs groups were almost the same (Figure 2C and Figure S3). Besides, co-staining of GFP and CD31 showed that only a few exogenous b-pEPCs directly differentiated into endothelial cells (Figure 2D), which did not match with the improved effect on vascular injury provided by b-pEPCs.

**Figure 3:**
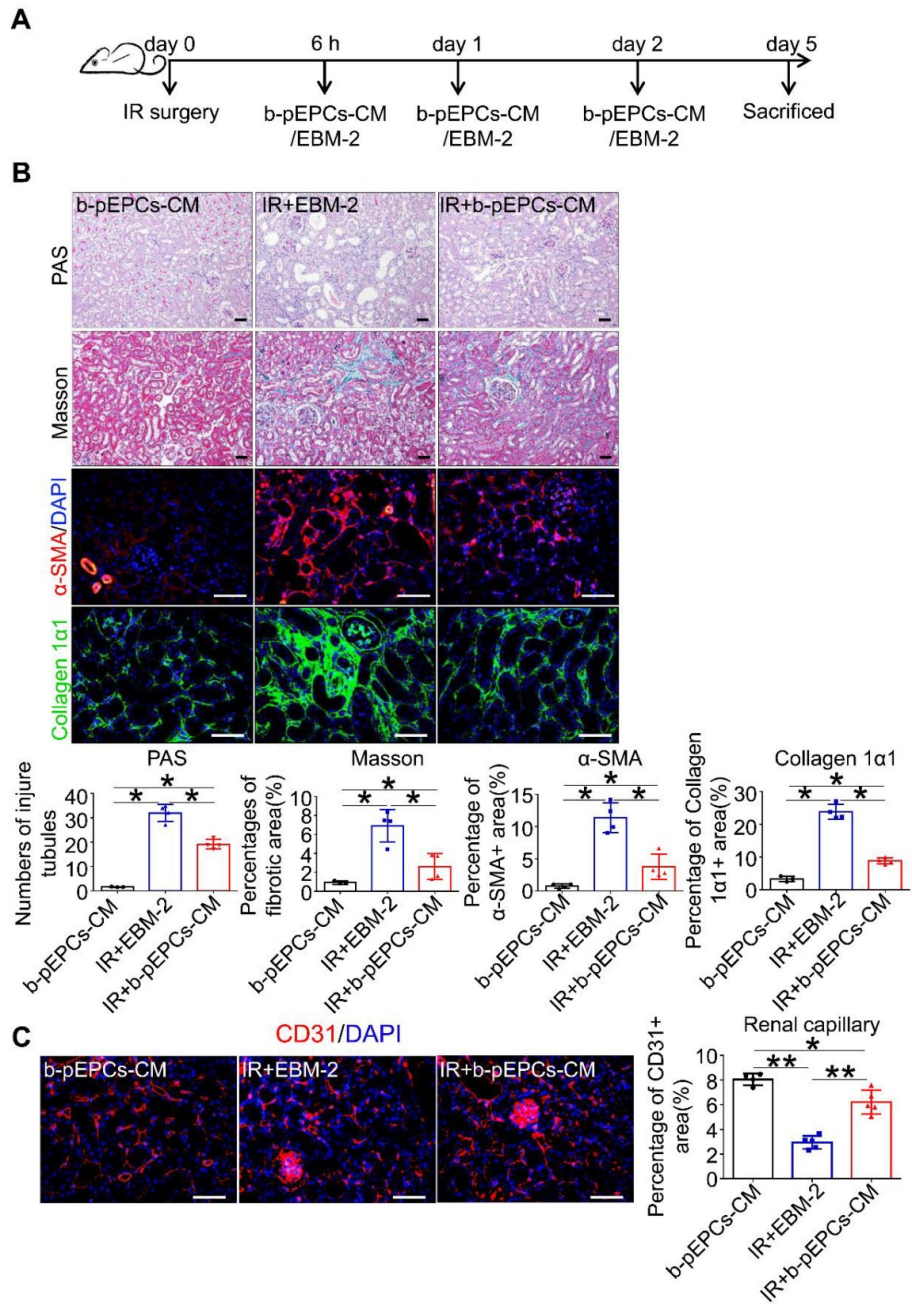
Exogenous b-pEPCs protected mice from IR-induced vascular injury and renal fibrosis via paracrine pathways.** A.** Schematic of mice treated with b-pEPCs-CM or vehicle medium EBM-2.** B.** PAS, Masson, α-SMA, and collagen 1α1 staining to evaluate renal injury and fibrosis.** C.** CD31 staining to visualize renal capillaries. Data are presented as the mean ± SD, n=3-5/group, ns: no significant difference, *p<0.05, **p<0.01. Scale bar: 50 μm.

Next, we collected b-pEPCs-CM, which was demonstrated to contain microvesicles that provided a protective effect in the UUO kidney 20. We injected b-pEPCs-CM collected from an equivalent dose of b-pEPCs (Figure 3A). The results showed a reduction in the number of injured tubules in IR+b-pEPCs-CM group as well as decreased fibrotic area. α-SMA staining showed more than 50% reduction in the IR+b-pEPCs-CM group compared with mice treated with the vehicle EBM-2 medium (IR+EBM-2 group). Also, collagen 1α1 was decreased in the IR+b-pEPCs group (Figure 3B). Immunofluorescence staining showed b-pEPCs-CM increased the number of CD31-positive endothelial cells in the IR+b-pEPCs-CM group (Figure 3C), which was confirmed by *in vitro* experiments in which b-pEPCs-CM inhibited H_2_O_2_-induced apoptosis in endothelial cells and preserved the tube formation (Figure S4). Furthermore, the administration of b-pEPCs and b-pEPCs-CM showed a comparable protective effect on IR-induced renal injury (Figure S5A and B). PAS and Masson staining also confirmed an equal improvement of tubular injury and interstitial fibrosis (Figure S5C). These data suggested that exogenous b-pEPCs protected IR-induced vascular injury and renal fibrosis via paracrine pathways.

### b-pEPCs culture medium improved IR-induced vascular injury and fibrosis by regulating PDGFR-β-positive pericytes

**Figure 4:**
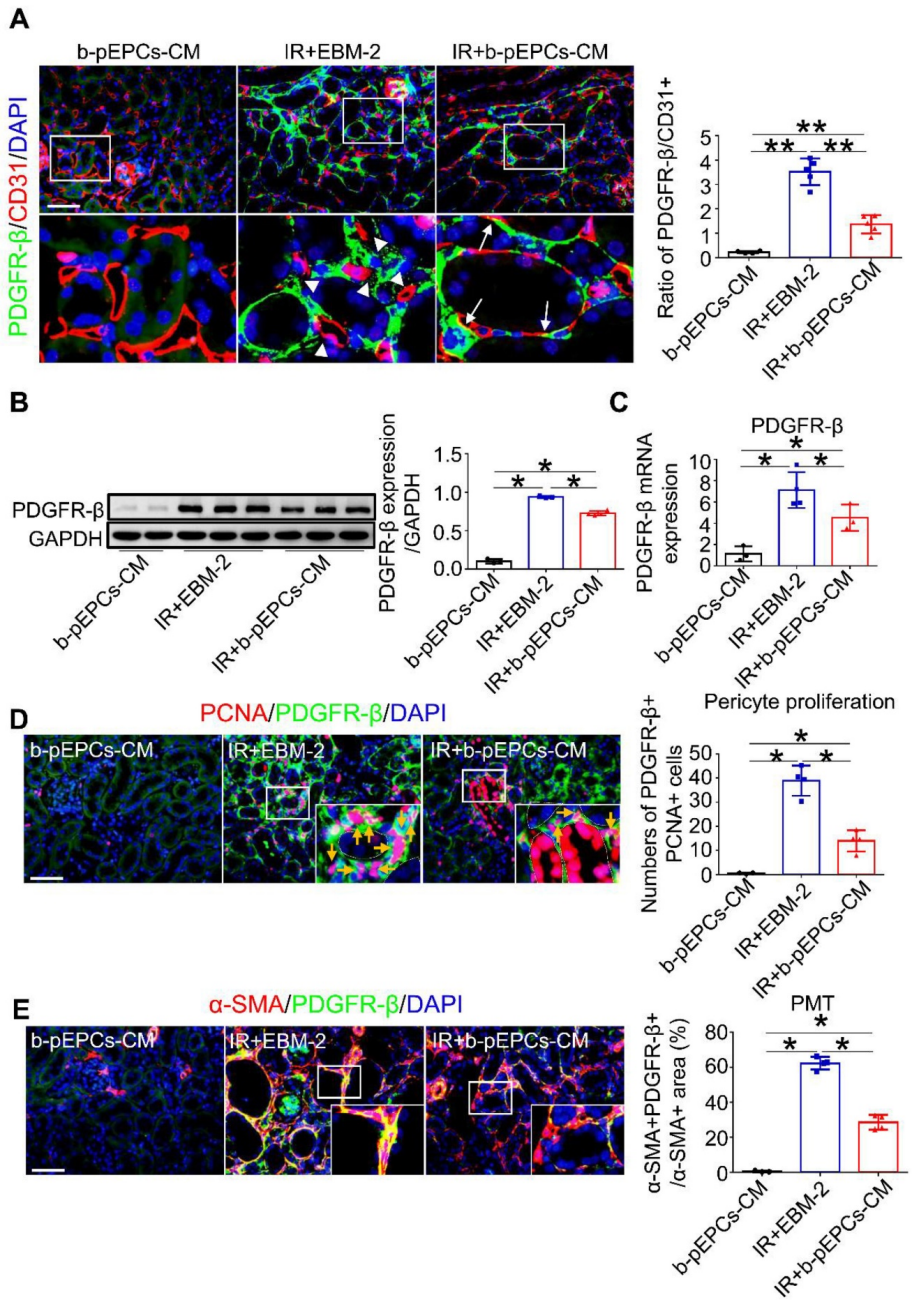
b-pEPCs improved IR-induced vascular injury and fibrosis by regulating PDGFR-β-positive pericytes. **A.** Immunofluorescence staining of PDGFR-β and CD31 to locate pericytes and endothelial cells; white triangles reveal the gap between pericytes and endothelial cells in the IR+EBM-2 group, white arrows represent pericytes attached to and located surrounding endothelial cells. The ratio of PDGFR-β/CD31 represents vascular structure stability and balance. In the normal kidney, PDGFR-β/CD31 is less than 0.4. The increased ratio implies abnormal and unstable vascular structures.** B and C.** Western blot and qRT-PCR to test PDGFR-β at protein and RNA levels. **D.** Co-staining of PCNA and PDGFR-β (orange arrows) to measure the number of proliferative pericytes.** E.** Co-staining of α-SMA and PDGFR-β to measure pericyte-derived myofibroblasts. The ratio of the α-SMA+PDGFR-β+/α-SMA+ area shows the portion of pericyte-derived myofibroblasts. Data are presented as the mean ± SD, n=3-5/group, *p<0.05, **p<0.01. Scale bar: 50 μm.

Immunofluorescence co-staining of PDGFR-β (a pericyte-specific marker) and CD31 showed only a small number of PDGFR-β-positive cells in the healthy kidney (Figure 4A). Numerous PDGFR-β-positive pericytes appeared in renal interstitium after IR on day 5 (IR+EBM-2 group) that were detached from CD31-positive endothelial cells. The histogram analysis showed a dramatically increased PDGFR-β/CD31 ratio, indicating microvascular structure disorder after IR surgery. In the IR+b-pEPCs-CM group, the PDGFR-β/CD31 ratio was higher than in healthy kidney but relatively lower than the IR+EBM-2 group. The re-attachment of PDGFR-β-positive cells to endothelial cells (CD31-positive) and the recovery of PDGFR-β/CD31 ratio demonstrated that secretory components from b-pEPCs improved the IR-induced microvascular structure disorder (Figure 4A).

We further explored the interaction of b-pEPCs with PDGFR-β-positive pericytes. Western blotting and qRT-PCR analyses showed that the expression of PDGFR-β was upregulated after IR injury, while it was attenuated by b-pEPCs-CM treatment (Figure 4B and C). Co-staining for PCNA and PDGFR-β indicated that IR led to the activation and proliferation of pericytes, which was inhibited by b-pEPCs-CM administration (Figure 4D). Co-staining for α-SMA and PDGFR-β revealed a high percentage (about 62.3%) of PDGFR-β-positive cells with high density expression of α-SMA after IR injury (IR+EBM-2 group). These results illustrated the differentiation of pericytes into myofibroblasts following renal injury 25, 26, which was reduced by b-pEPCs-CM injection (Figure 4E). Similar results were obtained by co-staining of PDGFR-β and collagen 1α1, another myofibroblast-specific marker (Figure S6). Thus, the secretary components from b-pEPCs could improve interstitial capillary rarefaction and sustain the microvascular structure stability following IR injury.

### b-pEPCs attenuated PDGFR-β-positive pericytes, vascular injury, and renal fibrosis

**Figure 5:**
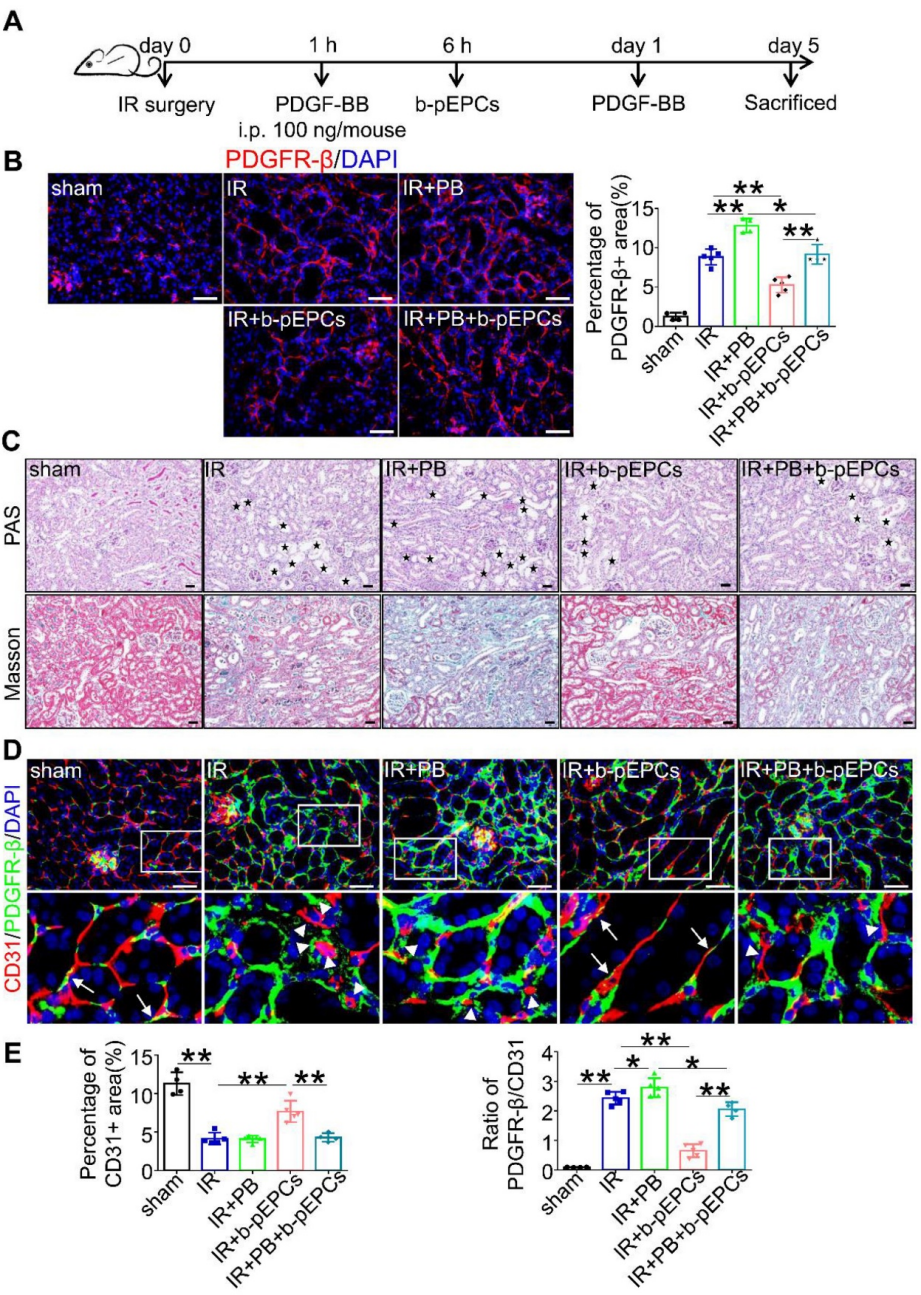
PDGF-BB (PB) increased the number of PDGFR-β-positive pericytes and exacerbated vascular injury and renal fibrosis, attenuated by b-pEPCs. **A.** Scheme of PDGF-BB treatment in mice. **B.** Measurement of PDGFR-β by immunofluorescence staining in different group. **C.** Histopathology staining to evaluate the influence of PDGF-BB on tubular injury and renal fibrosis. **D and E.** Co-staining of PDGFR-β and CD31 to evaluate the vascular structure. Data are presented as the mean ± SD, n=3-5/group, *p<0.05, **p<0.01. Scale bar: 50 μm.

PDGFR-β is a cell surface receptor of the platelet-derived growth factor (PDGF) family, including PDGF-AA, -BB, -CC, and -DD. The expression of PDGFR-β ligands in IR-induced renal injury measured by qRT-PCR analysis showed increased expressions of all ligands in the IR group with PDGF-BB showing a 3.31-times increase higher than PDGF-AA (1.36) and PDGF-DD (1.98) (Figure S7). Thus, we chose PDGF-BB as a stimulator of PDGFR-β (Figure 5A). Immunofluorescence staining showed that PDGF-BB further enhanced the expression of PDGFR-β caused by IR surgery. Exogenous b-pEPCs inhibited the expression of PDGFR-β in the IR+b-pEPCs group compared with the IR group (*p*=0.0040) and in the IR+PB+b-pEPCs group compared with the IR+PB group (*p*=0.0143) (Figure 5B). Histopathology staining revealed exacerbation of tubular injury and fibrotic collagen deposition in the IR+PB group. The exogenous b-pEPCs treatment resulted in a 45.8% reduction of tubular injury and 49.5% reduction of fibrosis area following PDGF-BB administration (IR+PB+b-pEPCs group) (Figure 5C and Figure S8A). The α-SMA and fibronectin staining showed PDGF-BB increased their expression following IR injury, which was inhibited by b-pEPCs administration (Figure S8B), indicating that PDGF-BB activated PDGFR-β-positive pericytes and accelerated renal fibrosis, which was attenuated by b-pEPCs treatment. CD31 immunostaining showed no difference between the IR and IR+PB groups (Figure 5D and 5E). However, the PDGFR-β/CD31 ratio increased (Figure 5E). The results implied that although PDGF-BB did not further worsen capillary rarefaction, it deteriorated the microvascular structure of pericyte-endothelial cells, while exogenous b-pEPCs improved interstitial capillary rarefaction and preserved microvascular structure.

### pericyte transition *in vitro*

**Figure 6:**
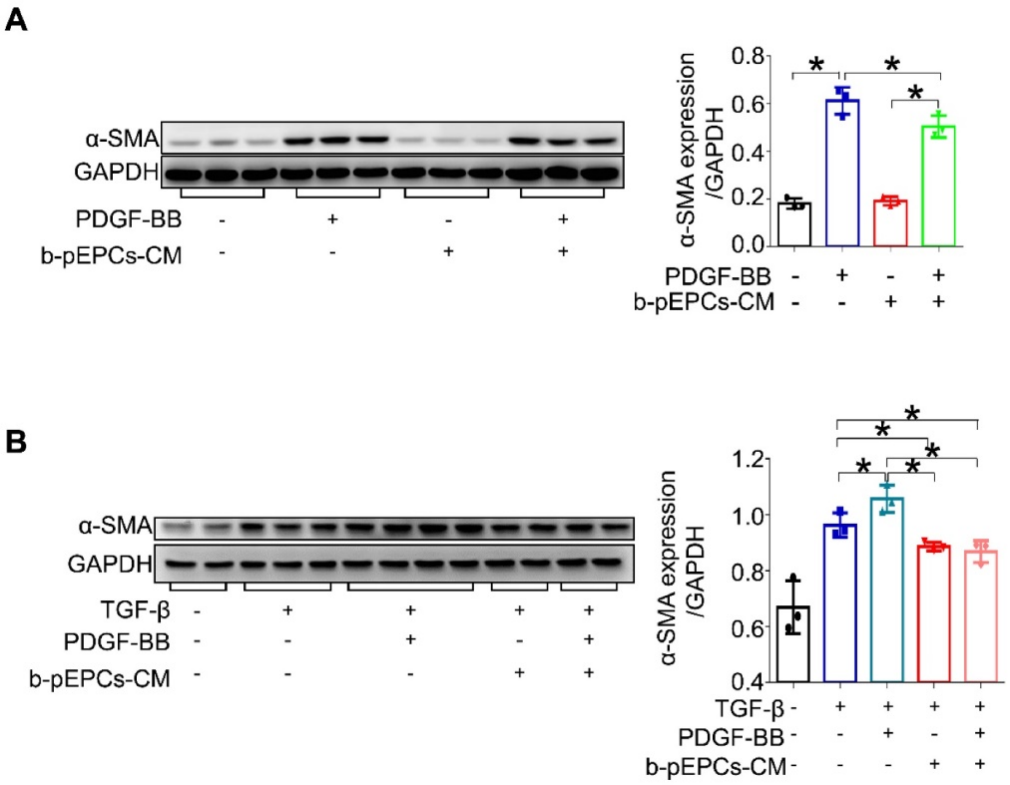
b-pEPCs inhibited PDGF-BB-induced pericyte transition *in vitro*.** A and B.** Pericytes were cultured *in vitro* and treated with PDGF-BB and/or TGF-β for 24 h. α-SMA expression was analyzed to evaluate the percentages of pericyte transition into myofibroblasts with different stimulators. Data are presented as the mean ± SD, n=3-4/group, *p<0.05.

To further elucidate the effect of b-pEPCs on pericytes, we cultured pericytes *in vitro* followed by stimulation with PDGF-BB, a profibrotic factor TGF-β, and culture medium from b-pEPCs. We found that both PDGF-BB and TGF-β upregulated α-SMA expression in pericytes, while b-pEPCs-CM blocked this effect (Figure 6), suggesting that the secretory components from b-pEPCs have the inhibitive ability on pericyte-myofibroblast transition.

### Exogenous b-pEPCs protection of vascular injury and renal fibrosis was blocked by PDGFR-β-positive pericyte depletion

**Figure 7:**
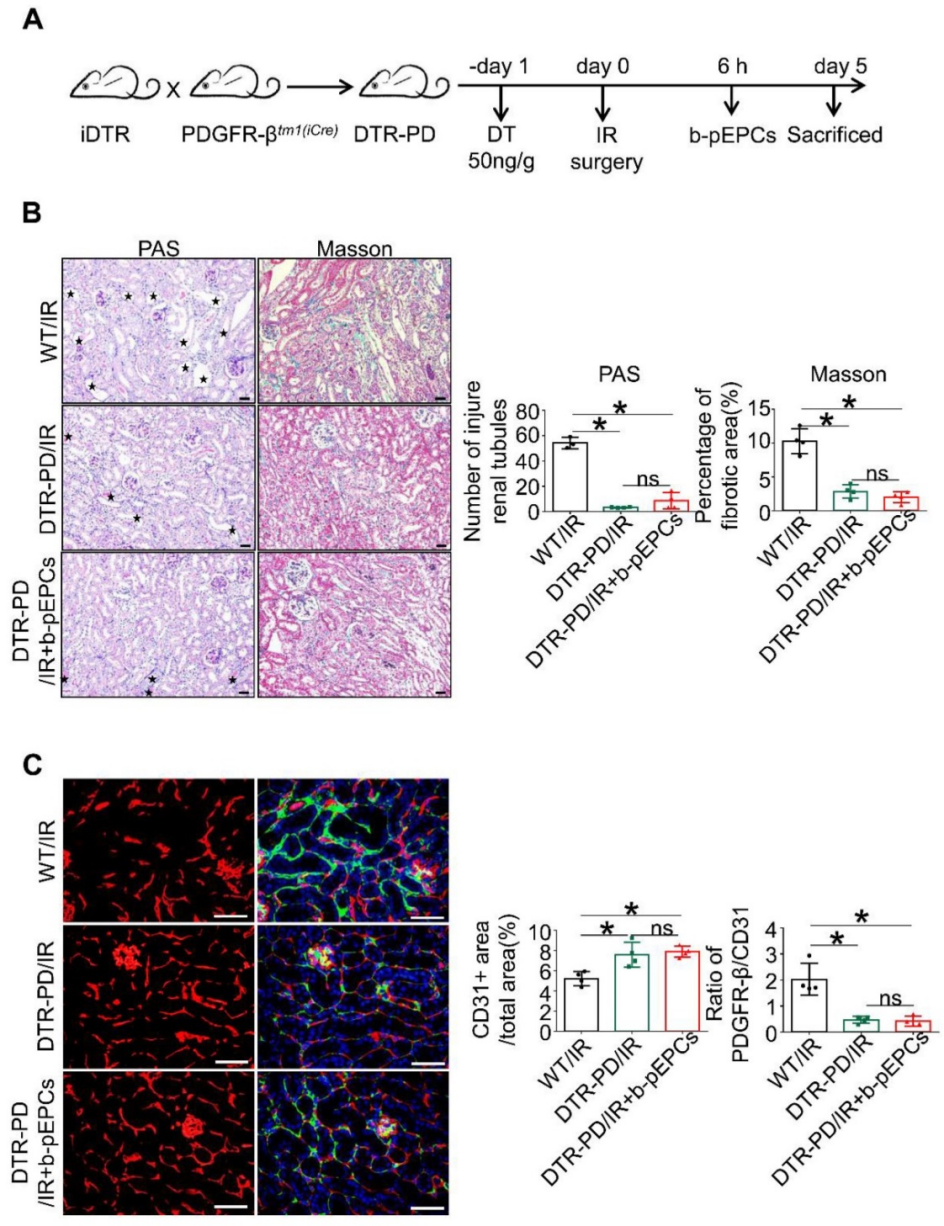
The protective effect of b-pEPCs on vascular injury and renal fibrosis was blocked by PDGFR-β-positive pericyte depletion. **A.** Scheme of the generation of selective PDGFR-β depleted mice (DTR-PD). **B.** Histopathology staining to evaluate the tubular injury and renal fibrosis after PDGFR-β deletion with DT. **C.** Co-staining of PDGFR-β and CD31 to test the influence of PDGFR-β depletion on renal vascular structures. Data are presented as the mean ± SD, n=4/group, ns: no significant difference, *p<0.05. Scale bar: 50 μm.

We generated selective PDGFR-β-positive cell depletion mice (DTR-PD) by crossing iDTR mice with PDGFR-β*^tm1(iCre)^* mice. After induction with DT, PDGFR-β-DTR-positive cells were depleted (Figure 7A). Western blotting and qRT-PCR analyses showed that in the DTR-PD/IR group, the expression of PDGFR-β decreased by 57.2% at the protein level and about 57.3% at the RNA level (Figure S9A and B). The tubular dilation, brush border loss, and interstitial fibrotic collagen deposition caused by IR at day 5 were improved by pericyte depletion (Figure 7B), indicating that the deletion of PDGFR-β-positive pericytes relieved IR-induced renal injury and early fibrosis. However, when comparing the DTR-PD/IR group with the b-pEPCs-treated control, we found that protective benefits of b-pEPCs on IR-induced tubular injury and early fibrosis were completely blocked (Figure 7B) confirmed by Western blotting (Figure S9C). Subsequently, we co-stained for CD31 and PDGFR-β and found that in the DTR-PD/IR group, the number of CD31-positive endothelial cells increased with attachment to PDGFR-β-positive pericytes and connected into networks (Figure 7C). In the DTR-PD/IR+b-pEPCs group, the vascular number and structure did not improve further (Figure 7C). Co-staining for PCNA with PDGFR-β and α-SMA with PDGFR-β showed an increase in both the number of PDGFR-β+PCNA+ and ratio of α-SMA+PDGFR-β+/α-SMA+ cells in the WT/IR group, whereas in the DTR-PD/IR group, the number of both proliferative and transformational pericytes decreased irrespective of the b-pEPCs treatment (Figure S9D). These results demonstrated that PDGFR-β-positive pericyte depletion ameliorated IR-induced vascular injury and renal fibrosis, and the protective effect of b-pEPCs on vascular injury and renal fibrosis was dependent on PDGFR-β-positive pericytes.

We further applied the DT 2 days after the IR surgery. The mice in the “DTR-PD/IR (DT injection before IR)” group were pre-injected with DT, while mice in the “DTR-PD/IR (DT injection 2 days after IR)” group were injected with DT after IR. The kidneys from the “DTR-PD/IR (DT injection 2 days after IR)” group showed decreased numbers of PDGFR-β-positive cells and increased density of fibrotic area when compared to the kidneys from the pre-injection group (Figure S10). These observations indicated that the transition of pre-existing PDGFR-β-positive pericytes into myofibroblasts is a pro-fibrotic process after IR injury and the existing fibrosis cannot be reverted by post-depletion of pericytes after IR. However, since the administration of b-pEPCs was before the post-depletion of pericytes, fibrosis was still inhibited in the post-depletion of pericyte group, similar to the group of “DTR-PD/IR (DT injection before IR)” (Figure S10), suggesting that the pericyte-derived myofibroblasts were activated and formatted in the early 2 days after IR injury. It also indicated that protection of acute ischemic injury. reduced renal fibrosis in DTR-PD mice.

## Discussion

It has been well established that differentiation of stem cells into endothelial cells rarely happens in healthy organs while occurring in injured organs at varying rates ranging from less than 0.1% to about 20% 27. Thus, previous studies focused on improving stem cell recruitment efficiency to target vessels or direct differentiation of stem cells into endothelial cells 28-31. Besides, stem cells were shown to use paracrine pathways, for example, through microvesicles for transferring proteins and RNAs to endothelial cells 9, 32-34. In Lerman's study autologous endothelial and/or mesenchymal progenitor cells improved renal microvasculature via regulating growth factors Ang-1, VEGF and attenuated renal inflammation etc., which have direct meaning to stem cells' effect on nephrology 35, 36. Our study demonstrated that b-pEPCs promoted vascular tube formation and improved IR-induced vascular injury. However, by employing different b-pEPCs tracking methods, we could not detect an adequate number of exogenous b-pEPCs in the injured kidney. These data indicated that mechanisms beyond direct endothelial differentiation play a role in exogenous b-pEPCs-induced vascular endothelial cell repair.

Pericytes are the mural cell types required for vessel maturation and functional maintenance around microvascular walls. In the vascular repair process, damaged endothelial cells are replaced by newly differentiated cells attached to vessel beds. Mural cells (pericytes in capillaries) surround and cover the endothelial layer in appropriate proportions through interactive communication to form integral functional vessels with adequate permeability 37, 38. The maturation phase of pericyte-endothelial cell attachment is consequential in vascular repair. Therefore, we tested microvascular structures in the injured kidney. Co-staining of CD31 and PDGFR-β showed noticeable gaps between pericytes and endothelial cells, indicating pericyte-endothelial cell detachment after IR injury. The ratio of PDGFR-β/CD31 was extremely small in healthy kidney and increased by more than 15-fold after IR. These results indicated that IR-induced vascular injury entailed a decreased number of endothelial cells and abnormal microvascular structure. Significantly, exogenous b-pEPCs administration narrowed the gaps between endothelial cells and pericytes, and the number of pericytes covering the endothelial layer returned to a normal level.

Our data suggested that b-pEPCs administration might preserve and remodel the microvascular structure. We further found that b-pEPCs administration inhibited the activation of PDGFR-β-positive pericytes, including their proliferation and transition into highly α-SMA-positive myofibroblasts. To further explore the relationship between b-pEPCs, pericytes, and IR-induced vascular injury and renal fibrosis, we used PDGFR-β-specific ligand PDGF-BB to inhibit pericyte activation. Data showed that compared with the IR group, PDGFR-β positive cells and the ratio of PDGFR-β/CD31 increased in the IR+PB (PDGF-BB) group, implying the activation of PDGFR-β-positive pericytes and the deterioration of the vascular structure integrity caused by PDGF-BB. Next, we deleted PDGFR-β-positive pericytes with DT injection and found that the vascular protection from b-pEPCs was blocked. These data supported that the protective effect of b-pEPCs on vascular injury and renal fibrosis was associated with the regulation of PDGFR-β-positive pericytes.

Besides regulating pericytes, we also examined b-pEPCs' effect on endothelial cells. The regulation of PDGFR-β-positive, pericyte-induced vascular structural stability, and endothelial cell-associated angiogenesis cooperated to promote vascular repair in the IR model. This notion was inconsistent with our observation in the previous study that b-pEPCs protected UUO-induced renal fibrosis without increasing capillary distribution 20. We suspected the insufficient vascular repair capacity might be attributed to the more dilated tubules in the UUO model than in the IR model, leading to significantly limited interstitial space for capillaries.

The present study provided a new insight into how hematopoietic stem cells affected vascular injury; besides regulating endothelial cells, b-pEPCs can potentially remodel and stabilize capillary structures by interacting with pericytes. We also compared b-pEPCs with b-pEPCs-CM and found they had an equal protective effect on IR-induced renal fibrosis, demonstrating the b-pEPCs-instigated paracrine mechanism. b-pEPCs probably regulate PDGFR-β-positive pericytes via crosstalk through secretion of growth factors, transferring mRNA, or cellular trafficking. However, there were still some limitations. First, b-pEPCs are hematopoietic stem cells, defined as premature-endothelial cells that are capable of differentiating into endothelial cells. Lack of a specific marker cause difficulties in exploration and investigating b-pEPCs. We adopted most commonly used markers of b-pEPCs and functional identification with Ac-LDL and UEAI staining in present study. But the sharing of several common characteristics between b-pEPCs and other hematopoietic cells such as CD34 cause difficulties to separate b-pEPCs from other bone marrow derived progenitor cells. More study should be done to purify b-pEPCs for future investigation. Second, intra-renal pericytes act not only as mural cells, but also a resource of myofibroblasts. Whether b-pEPCs regulated endothelial cell-pericyte detachment, and the subsequent proliferation and transition, or b-pEPCs directly triggered the switch of pericyte from resting to activated status remains unknown. Besides, we did not detect the proteomics and transcriptomics of b-pEPCs and pericytes and did not analyze b-pEPCs ligand-pericyte receptor pairs, to assess the exact paracrine mechanism of b-pEPCs on pericytes, which needs to be figured out in future.

In summary, we provided evidence for the protective effect of b-pEPCs on vascular injury and showed that early renal fibrosis was dependent on PDGFR-β-positive pericytes. Whether the PDGF-BB neutralization could provide additional renal protection needs to be further explored.

## Conclusion

In the present study, we demonstrated that exogenous b-pEPCs alleviate IR-induced vascular injury and early renal fibrosis, which depends on PDGFR-β-positive pericytes via a paracrine mechanism.

## Supplementary Material

Supplementary figures.

## Figures and Tables

**Table 1:** Sequences of primers used for qRT-PCR.

Gene	Primer
Mouse-GAPDH	5'-TTGATGGCAACAATCTCCAC-3'; 3'-CGTCCCGTAGACAAAATGGT -5'
Mouse-α-SMA	5'-GTCCCAGACATCAGGGAGTAA-3'; 3'-TCGGATACTTCAGCGTCAGGA-5'
Mouse-Fibronectin	5'-GCTCAGCAAATCGTGCAGC-3'; 3'-CTAGGTAGGTCCGTTCCCACT -5'
Mouse-PDGFR-β	5'-CGGATGTCACTGAGACGACAA-3'; 3'-AGTAAGTATCGGAGTCCACTTCC-5'

## Abbreviations

pEPCs: putative endothelial progenitor cells
b-pEPCs: bone marrow derived-pEPCs
IR: ischemia-reperfusion
PDGFR-β: platelet derived growth factor receptor β
PDGF-BB: PDGF chain B
ECM: extracellular matrix
CKD: chronic kidney disease
UUO: unilateral ureteral obstruction
WT: wild type
pEPCs-CM: pEPCs-cultured medium
DT: diphtheria toxin
HBVP: human brain vascular pericytes
PM: Pericyte Medium
BMT: bone marrow transplantation
BMCs: bone marrow-derived cells
EGFP: CAG-enhanced green fluorescent protein
PBS: phosphate-buffered saline
TGF-β: transforming growth factor-β
PAS: periodic acid Schiff
α-SMA: α-smooth muscle actin
PCNA: proliferative cell nuclear antigen
DAPI: 4′6-diamidino-2-pheny-lindole
BUN: blood urea nitrogen
HUVECs: human umbilical vein endothelial cells
SDF-1: stromal cell-derived factor-1
PMT: pericytes transitioning into myofibroblasts
