# Supplementary material for: Exogenous bone marrow derived-putative endothelial progenitor cells attenuate ischemia reperfusion-induced vascular injury and renal fibrosis in mice dependent on pericytes
Source: Theranostics. 2020 Oct 25;10(26):12144–57. doi: 10.7150/thno.48562 (PMC7667688; doi:10.7150/thno.48562)
Supplement: Supplementary file 1 — Supplementary figures. [file thnov10p12144s1.pdf]

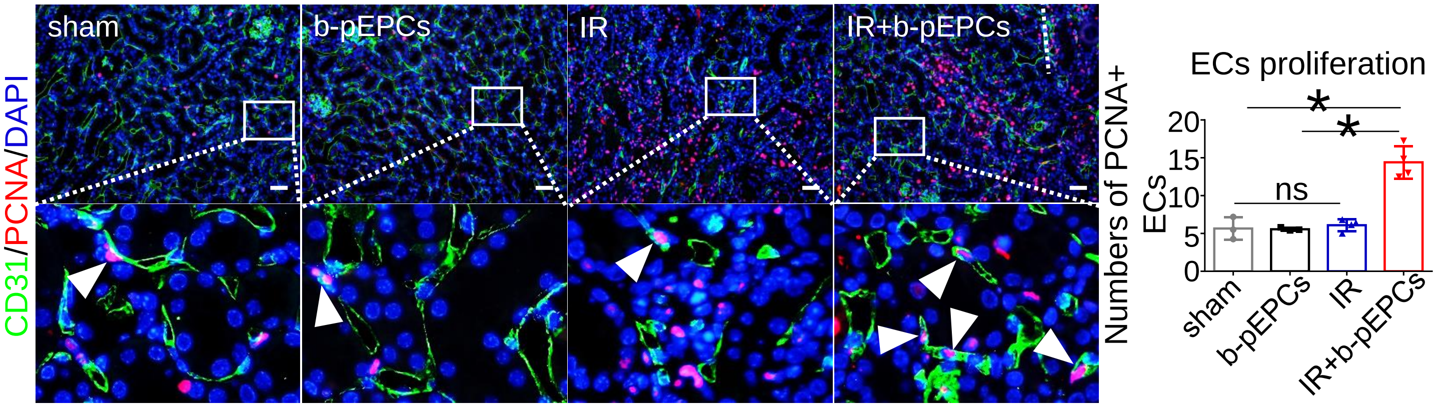

Figure S1. b-pEPCs promoted angiogenesis in vivo. Co-staining of CD31 and PCNA to measure proliferative endothelial cells (white triangles). In healthy kidney, endothelial cells are mostly at resting status, only a few cells keep proliferating. IR led to endothelial cell apoptosis and vascular injury, which activate endothelial cells for self-repair, the histogram showed that on IR-day5, the proliferative endothelial cells in the IR group was at the same level as in sham groups. In IR+b-pEPCs group, they were still at a rather higher proliferative state. The data were presented as the mean ± SD, n=3-4/group, *p<0.05, **p<0.01, ns: no significant difference. Scale bar: 50 μm.

A

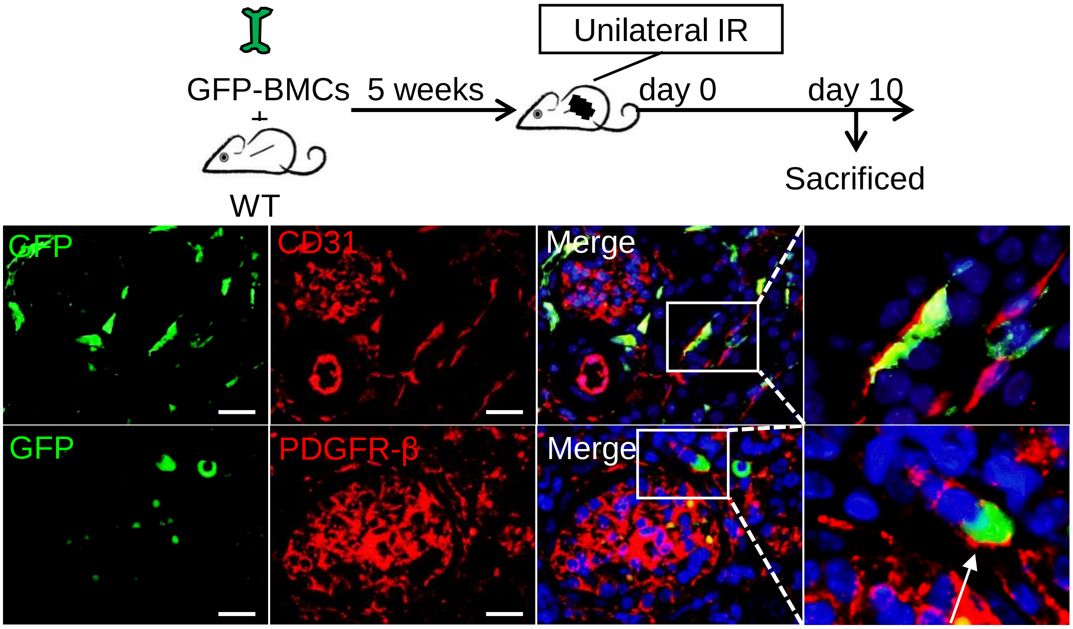

B

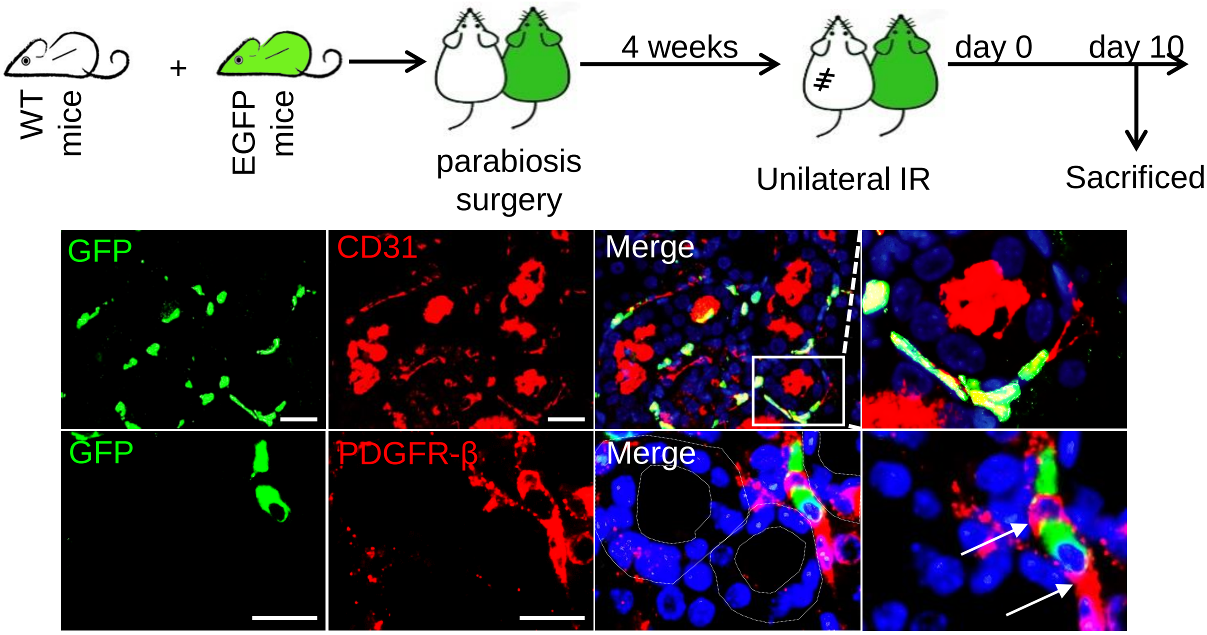

Figure S2. Bone marrow-derived cells (BMCs) differentiated into endothelial cells and participated in angiogenesis. **A.** The scheme of bone marrow transplantation (BMT). GFP-labelled BMCs were tracked in IR operated mice. Co-staining of GFP and CD31 meant that exogenous BMCs expressed CD31 in injured kidney. Co-staining of GFP and PDGFR-β presented GFP positive b-pEPCs located attached with PDGFR-β positive pericytes. **B.** The scheme of conjoined parabiosis. Co-staining showed that cells from healthy mice were recruited to the injured kidney and expressed endothelial cell marker CD31. Co-staining of GFP and PDGFR-β implied the location of exogenous b-pEPCs was close to pericytes. Both results implied that BMCs owned the capacity of differentiation into endothelial cells and participated in angiogenesis. Scale bar: 20 μm.

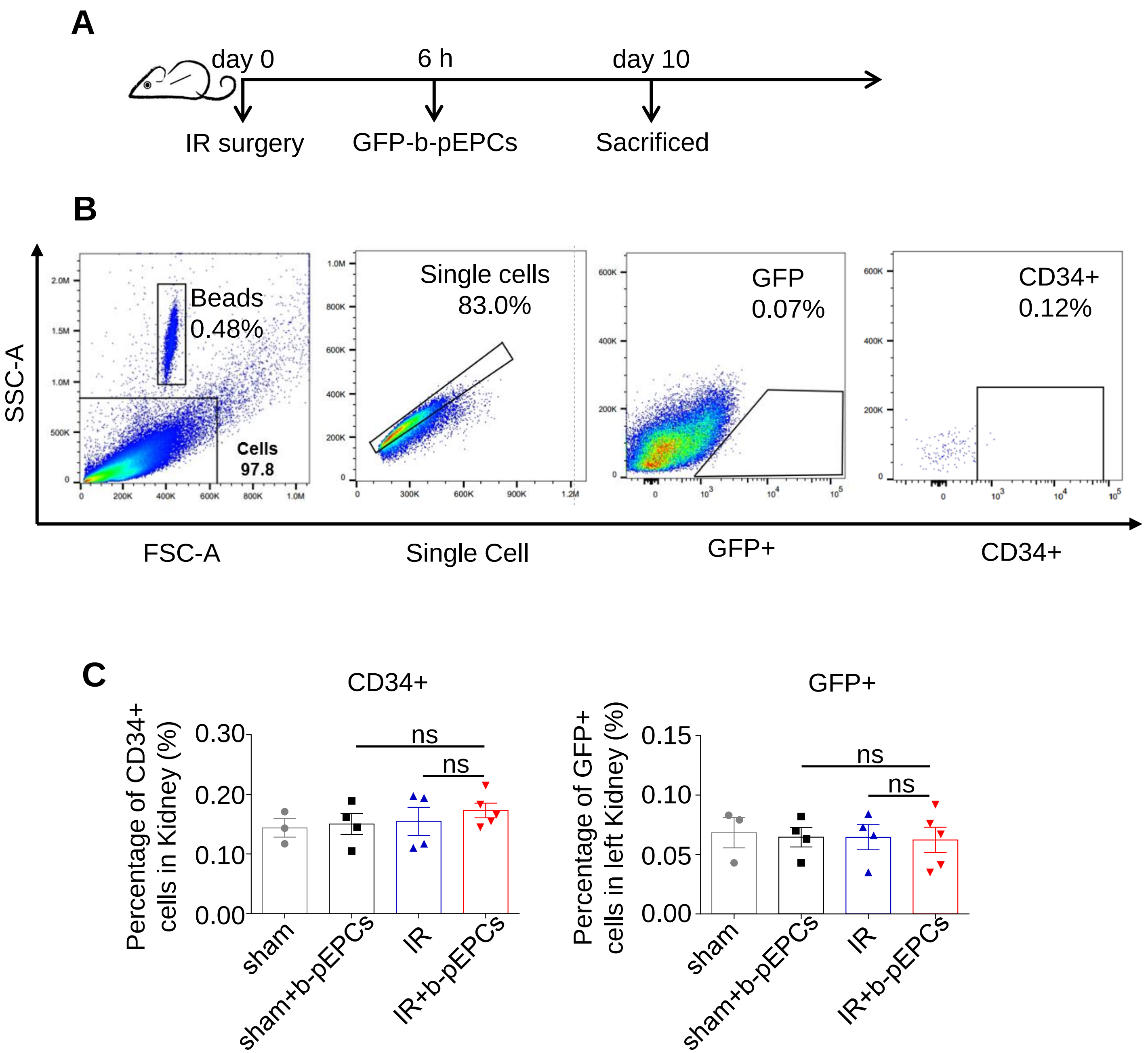

Figure S3. Tracking of b-pEPCs in injured kidney. **A.** The Scheme of this part. **B.** The gating strategy and images from cytometer. **C.** b-pEPCs isolated from EGFP mice were transplanted into mice after IR surgery. b-pEPCs calculation was measured in IR-day 10. Both CD34-positive cells and GFP-positive cells were counted by flow cytometry to track exogenous b-pEPCs in IR-operated kidney. The data were presented as the mean ± SD, n=3-5/group, *p<0.05, ns: no significant difference.

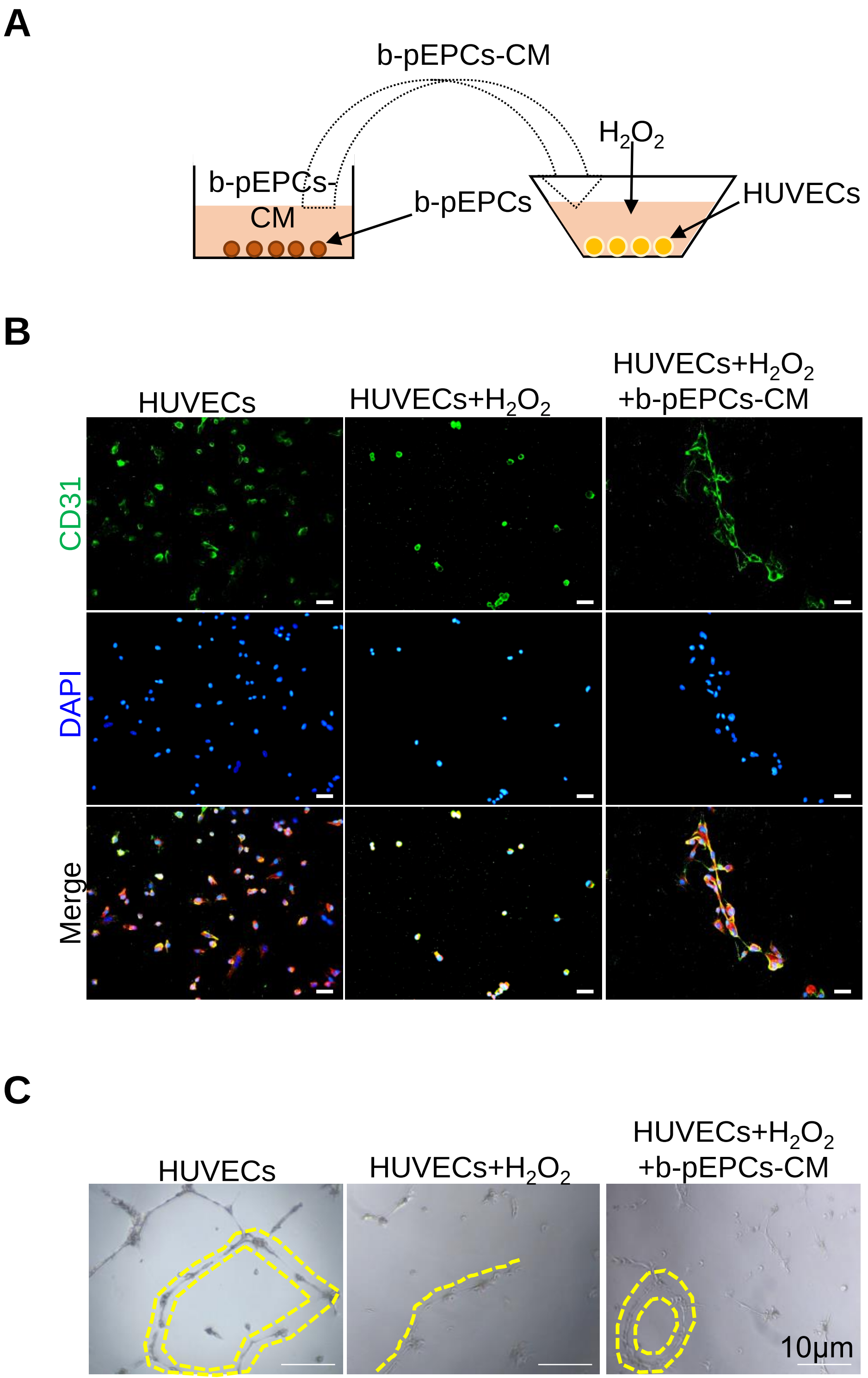

Figure S4. b-pEPCs promoted angiogenesis in vitro. **A.** Scheme of culture and stimulation of human umbilical vein endothelial cells (HUVECs) in vitro. **B.** immunofluorescent staining of HUVECs. In HUVECs+H_2O_2 group, the number of endothelial cells significantly decreased compared to HUVECs group. With the b-pEPCs-CM treatment, the number increased, and cells distributed in tube formation shape. Scale bar: 20μm. **C.** 3D-tube formation test showed endothelial cells formatted in tube shape. H_2O_2 damaged this ability and cells scattered in the Matrigel. In HUVECs+H_2O_2+b-pEPCs-CM group, part of the cells preserved the tube formation ability. Scale bar: 10 μm. The data were presented as the mean ± SD, n=3-4/group, *p<0.05.

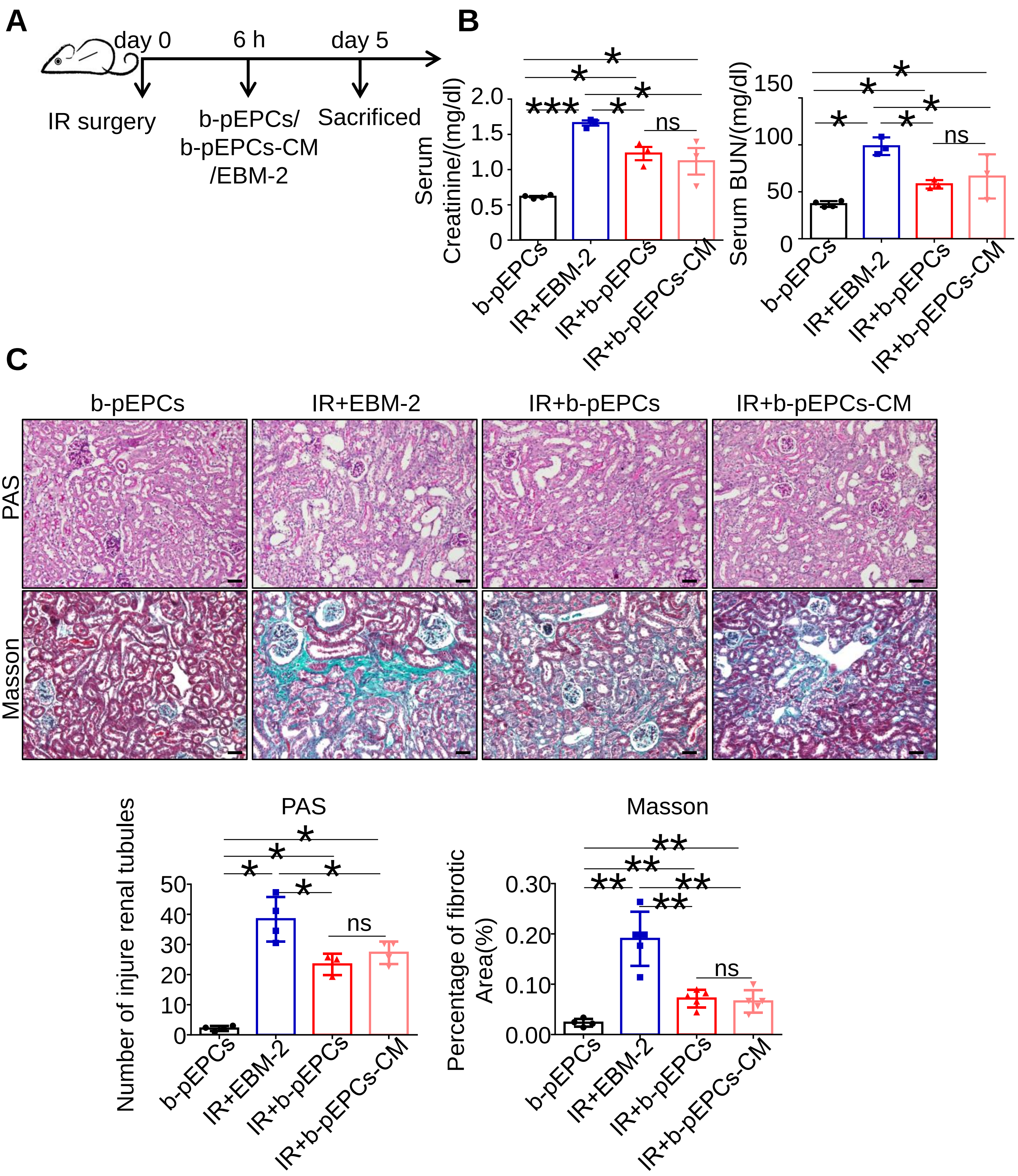

Figure S5. b-pEPCs-CM had an equivalent protective effect on IR-induced renal injury and fibrosis as b-pEPCs. **A.** The Scheme of this part. **B.** Serum BUN and creatinine detected by biochemical kit. **C.** Pathological staining and statistical histograms. The data were presented as the mean ± SD, n=4-5/group, ns: no significant difference, **p*<0.05, ***p*<0.01. Scale bar: 50 μm.

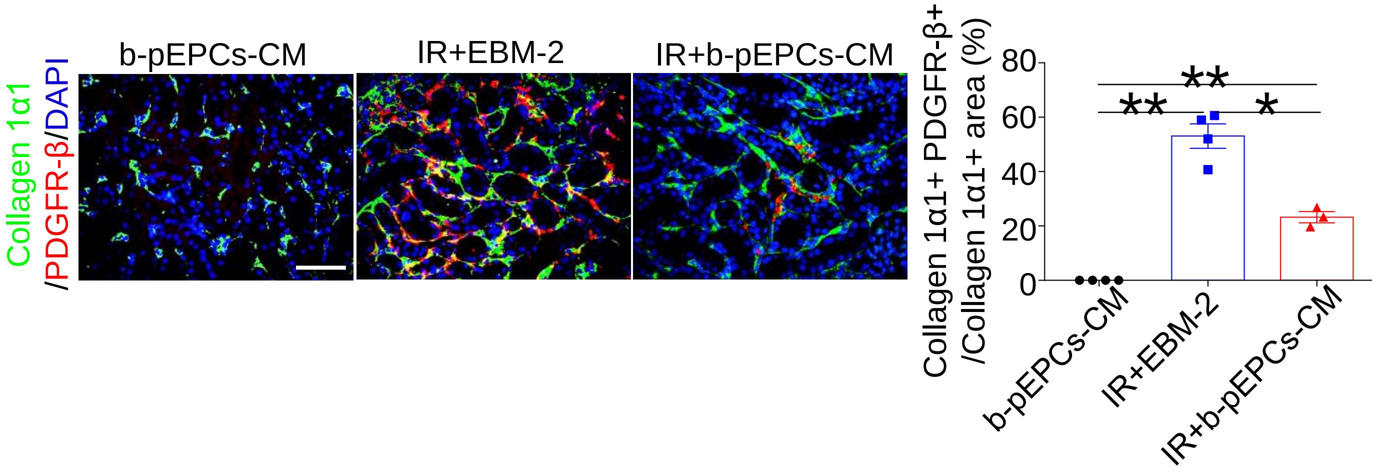

Figure S6. Pericyte-myofibroblast transition. Co-staining of collagen 1α1 and PDGFR-β to measure pericyte-derived myofibroblast. The ratio of collagen 1α1+PDGFR-β+/collagen 1α1+ area was to further calculate the portion of myofibroblast derived from pericyte. The data were presented as the mean ± SD, n=4/group, *$p<0.05$, **$p<0.01$. Scale bar: 50 μm.

qRT-PCR

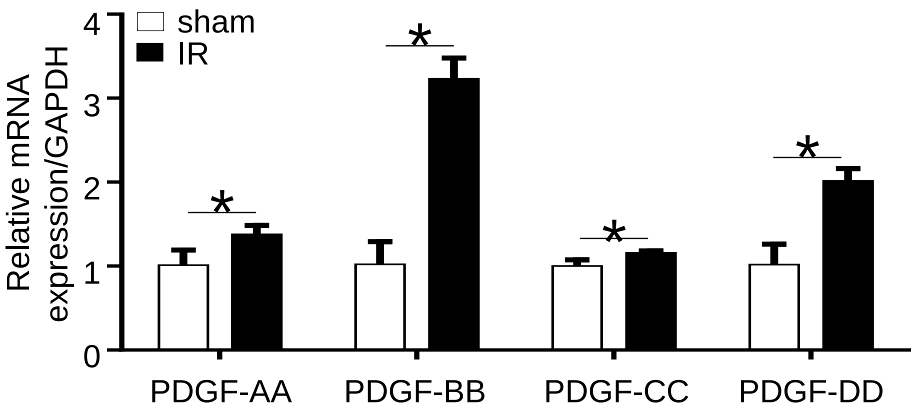

Figure S7. IR increased the expression of PDGFs. Analysis of PDGFR-β' ligands by qRT-PCR. The data were presented as the mean ± SD, n=3-5/group, *p<0.05.

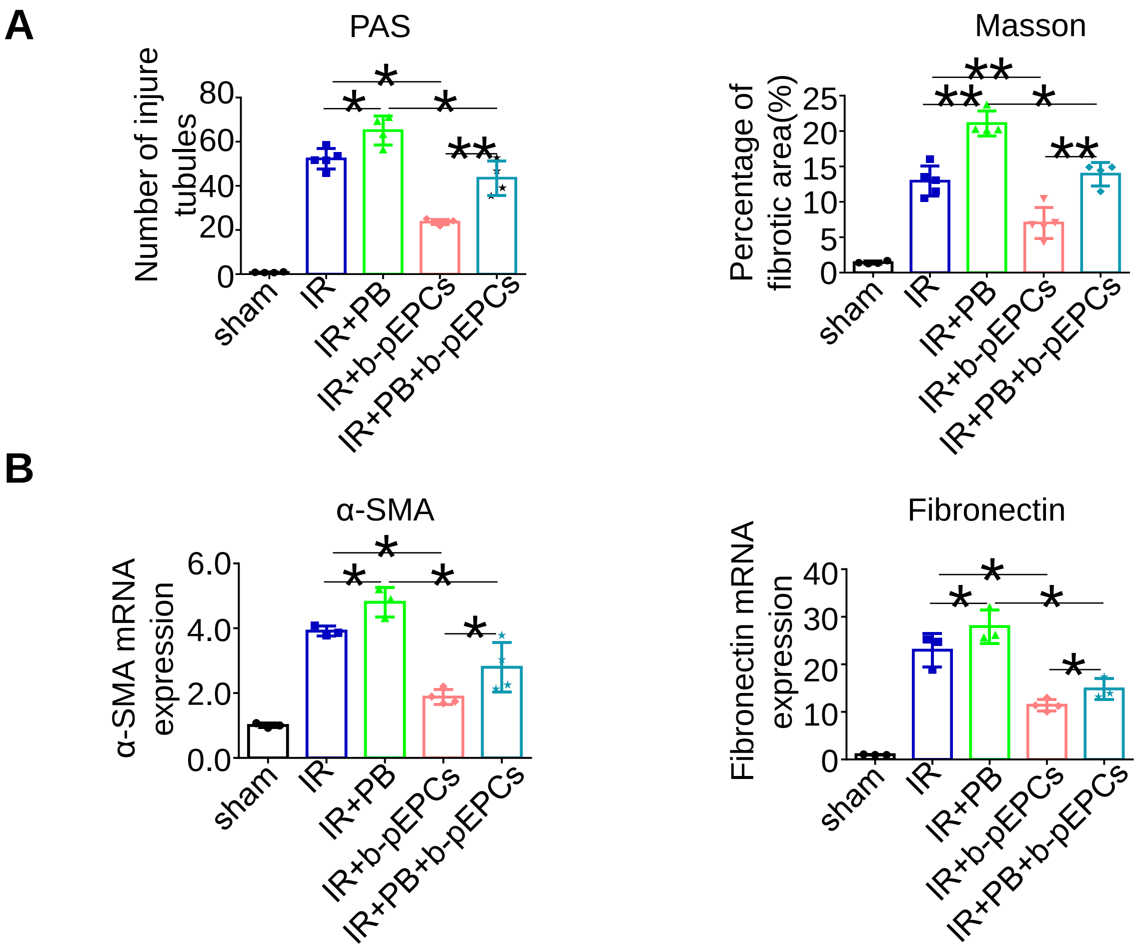

Figure S8. PDGF-BB increased the expression of PDGFR-β positive pericytes and exacerbated renal fibrosis, which was ameliorated by b-pEPCs. **A.** Histogram of PAS and Masson staining in Figure 5C. **B.** The measurement of fibrotic markers α-SMA and Fibronectin in RNA level by qRT-PCR. The data were presented as the mean ± SD, n=3-5/group, *p<0.05, **p<0.01.

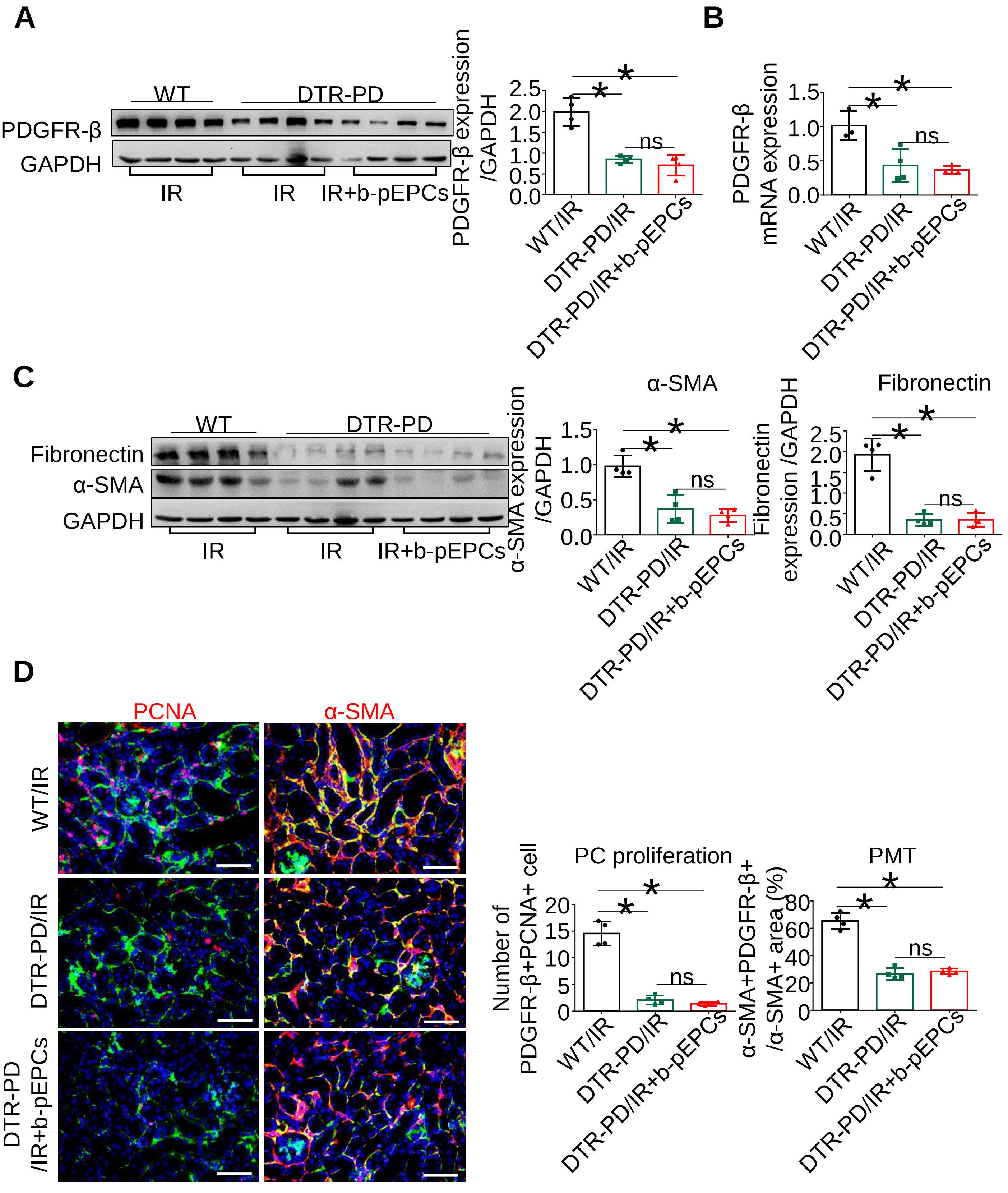

Figure S9. The protective effect of b-pEPCs on vascular injury and renal fibrosis was blocked by PDGFR-β positive pericyte depletion. **A and B.** Western blot and qRT-PCR to analyze PDGFR-β expression. **C.** Assessment of renal fibrosis with fibrotic markers α-SMA and Fibronectin in protein level. **D.** Co-staining of PDGFR-β with PCNA and α-SMA to check pericyte status of proliferation and transition. The data were presented as the mean ± SD, n=4/group, ns: no significant difference, *p<0.05. Scale bar: 50 μm.

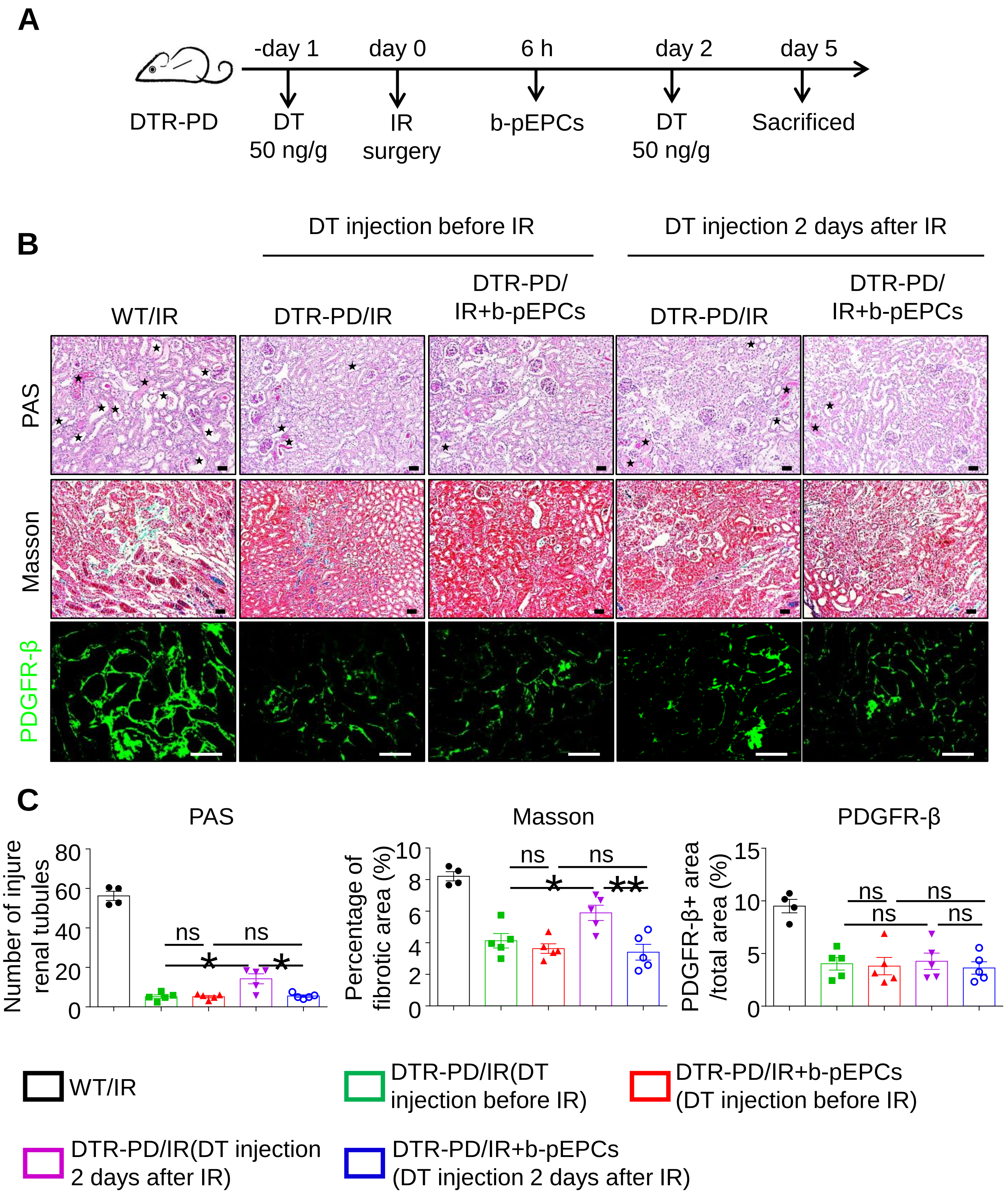

Figure S10. DT deleted PDGFR-β positive pericyte and ameliorated pericyte-myofibroblast transition. Existed fibrosis can't be reverted by DT injection 2 days after IR surgery**. A.** The scheme of this part. **B and C.** Pathological staining (PAS and Masson) to evaluate tubular injury and renal fibrosis in DTR-PD mice with or without DT injection before or after IR surgery. PDGFR-β was measured to observe the number of pericyte. The data were presented as the mean ± SD, n=3-5/group, ns: no significant difference, *p<0.05, **p<0.01. Scale bar: 50 μm.
